# Rapid Detection and Quantification of Patulin and Citrinin Contamination in Fruits

**DOI:** 10.3390/molecules26154545

**Published:** 2021-07-27

**Authors:** Sudharsan Sadhasivam, Omer Barda, Varda Zakin, Ram Reifen, Edward Sionov

**Affiliations:** 1 Department of Food Sciences, Institute of Postharvest and Food Sciences, Agricultural Research Organization, Volcani Center, Rishon LeZion 7528809, Israel; sudharsan@volcani.agri.gov.il (S.S.); barda@volcani.agri.gov.il (O.B.); veredz@volcani.agri.gov.il (V.Z.); 2 The Robert H. Smith Faculty of Agriculture, Food and Environment, Institute of Biochemistry, Food Science and Nutrition, The Hebrew University of Jerusalem, Rehovot 7610001, Israel; ram.reifen@mail.huji.ac.il

**Keywords:** mycotoxin detection, pome fruits, patulin, citrinin, HPLC

## Abstract

Patulin (PAT) and citrinin (CTN) are the most common mycotoxins produced by *Penicillium* and *Aspergillus* species and are often associated with fruits and fruit by-products. Hence, simple and reliable methods for monitoring these toxins in foodstuffs are required for regular quality assessment. In this study, we aimed to establish a cost-effective method for detection and quantification of PAT and CTN in pome fruits, such as apples and pears, using high-performance liquid chromatography (HPLC) coupled with spectroscopic detectors without the need for any clean-up steps. The method showed good performance in the analysis of these mycotoxins in apple and pear fruit samples with recovery ranges of 55–97% for PAT and 84–101% for CTN, respectively. The limits of detection (LOD) of PAT and CTN in fruits were 0.006 µg/g and 0.001 µg/g, while their limits of quantification (LOQ) were 0.018 µg/g and 0.003 µg/g, respectively. The present findings indicate that the newly developed HPLC method provides rapid and accurate detection of PAT and CTN in fruits.

## 1. Introduction

Mycotoxins are a group of natural secondary metabolites produced by various filamentous fungi. These toxic compounds contaminate raw agricultural commodities, foods, and feeds either before harvest or under poor postharvest management. Exposure to mycotoxins can pose a serious health threat to both humans and animals [1]. The adverse health effects range from long-term chronic conditions, such as immune deficiency and cancer, to acute poisoning leading to organ failure [2]. Patulin (PAT) is a polyketide lactone mycotoxin that was first isolated in the 1940s from *Penicillium patulum* [3] but is also produced by numerous *Penicillium*, *Aspergillus,* and *Byssochlamys* species [4]. *Penicillium expansum* (blue mold) is the main source of PAT contamination in infected (deteriorated) apples and apple-based products such as juices, jams, and ciders. Other fruits found contaminated with PAT include pears, peaches, apricots, plums, strawberries, and kiwifruits [5,6,7]. PAT is reported to affect kidney cells [8]. Moreover, PAT possesses neurotoxic, immunotoxic, mutagenic, and carcinogenic properties [9,10,11,12]. Due to its toxicity and adverse health effects, a provisional maximum tolerable daily intake of PAT for humans has been established to be 0.4 µg/kg body weight [13]. The European Commission Regulation 1881/2006 has established the maximum allowed levels for PAT in different foodstuff [14]. According to the regulation, the maximum acceptable level of PAT must not exceed 50 µg/l in fruit juices, spirit drinks, and ciders, 25 µg/kg in solid apple products, and 10 µg/kg in food intended for infants and young children. Citrinin (CTN) is a hepatotoxic and nephrotoxic mycotoxin, first isolated from *Penicillium citrinum* [15] but also produced by other members of the genus *Penicillium*, as well as by several *Monascus* and *Aspergillus* species [16]. CTN can be found as a contaminant in long-stored foods such as grains and cereal-based products and also in fruits, herbs, spices, and moldy cheeses [16,17,18,19]. Limited data concerning CTN toxicity are available in the literature. Several studies have shown that exposure to CTN induces nephrotoxicity, hepatotoxicity, and chromosomal abnormalities in experimental animal models [20,21,22,23]. It has been reported that similar to ochratoxin A, CTN was also implicated as a potential risk factor for human Balkan endemic nephropathy [24]. However, this mycotoxin is classified in group 3 by the International Agency for Research on Cancer as not classifiable as to its carcinogenicity to humans, because of limited evidence in animals [25].

Monitoring PAT and CTN becomes critical for maintaining high-quality foodstuffs and for control of the mycotoxins levels in different commodities to ensure food safety. Therefore, there is a need for further development of rapid and accurate methods for PAT and CTN detection. In general, qualitative analysis of mycotoxins requires sample clean-up, followed by analysis with high-performance liquid chromatography (HPLC) and detection using UV/fluorescence or photodiode array detector. In recent years, there is a strong trend toward multi-mycotoxin testing with high sensitivity and low detection limit using advanced LC-MS/MS instruments. Although LC-MS/MS-based methods are quick and sensitive, they are not widely available because of high analysis and maintenance costs. Therefore, conventional HPLC methods with various sample cleaning processes are routinely developed for mycotoxin detection and quantification.

The success of extraction and analysis of mycotoxins depends on their chemical nature and the matrix complexity in which they are dispersed. In the analytical process of PAT and CTN, sample preparation and pretreatment are crucial steps requiring skilled manual work for the accurate analysis of the target compound [26,27]. The extraction and analysis of PAT are more challenging than CTN due to PAT’s low molecular weight and high polar nature. Thus, the efficiency of extraction and clean-up steps improves the sensitivity, precision, and specificity of the method. In the last decade, various sample preparation methods based on solid-phase extraction (SPE) were developed for the analysis of PAT and CTN in fruits and fruit products [26,28,29]. The major drawback of the SPE procedure is that it is time consuming, especially when large numbers of samples need to be analyzed [30]. Immunoaffinity columns (IACs) have been widely used for mycotoxin clean-up in complex matrices prior to chromatographic analysis due to their high degree of selectivity and specificity. An HPLC method based on IAC clean-up has been already developed to determine CTN in different matrices [31]. The fact that these columns are only used once and their relatively high cost are major disadvantages. The use of various modifications of the QuEChERS methodology (acronym of quick, easy, cheap, effective, rugged, and safe) has also been introduced for optimized extraction of PAT from fruit samples [7,32,33]. However, the QuEChERS procedure requires subsequent clean-up of the obtained extract, which increases the cost of the analysis [34]. The purpose of the current study was to develop an accurate and commonly available analytical procedure for PAT and CTN analysis in relatively complex fruit matrices, such as apples and pears, in order to evaluate the natural occurrence of these mycotoxins in solid pome fruits. Therefore, simple and reliable extraction methods have been developed for PAT and CTN determination by HPLC, coupled with spectroscopic detectors in apples and pears.

## 2. Results and Discussion

### 2.1. Method Validation

HPLC chromatograms of spiked PAT and CTN were very clear when compared to the blank samples (Figure S1). The peaks obtained for both toxins were separated from each other without any interferences from the sample matrices, demonstrating the specificity of the procedure. PAT and CTN were separated at 3.6 and 3.7 min, respectively. Hence, the selectivity and specificity of both mycotoxin were considered satisfactory.

The linearity of spiked standard mycotoxins in yeast extract sucrose (YES) agar medium and fruits was assessed by linear regression analysis based on eight calibration points in the range of 0.005–50 µg/g. The calibration curves calculated for both PAT and CTN in all matrices were considered to be linear in the studied range, with an average correlation coefficient equal to 0.999 (Figure S2).

The limits of detection were calculated as the lowest concentration resulting in a response of three times the average of the baseline noise obtained from diluted spiked blank samples. The limits of quantification were defined as three times greater than the LOD values. Results for LOD and LOQ are summarized in Table 1.

Recovery studies were carried out by spiking blank samples of YES agar medium and fresh fruits with known concentrations of standard mycotoxins. The mean recoveries of PAT and CTN in synthetic medium ranged from 57% to 93% and 71% to 95% with the relative standard deviation (RSD) range of 0.67–4.7% and 0.85–6.95%, respectively, within the spiking levels of 0.005–50 µg/g (Table 2). According to the Commission Regulation guidelines [35], PAT recovery in fruits was within the acceptable range, which is 55–97%. Since PAT is more stable in a slightly acidic environment than in a basic medium, the extraction procedure was carried out at acidic pH to avoid degradation of the mycotoxin. The advantage of keeping an acidic pH during the extraction, which resulted in a relatively high PAT recovery, has been previously observed in several studies [7,36,37]. Although there are no specific regulations or guidelines for CTN, the current study found high recovery rates of this mycotoxin in fruits (84–101%), consistent with the results obtained in previous studies [31,38,39]. The high CTN recovery in fruits was achieved due to efficient extraction using 100% methanol only, compared to other studies where extraction of CTN with methanol involved a further cleanup step with SPE or IAC columns [31,40]. The RSD values obtained for each mycotoxin at different spiking levels were below 10%, showing good precision for both apple and pear matrices (Table 2).

### 2.2. Analysis of Samples

**Figure 1:**
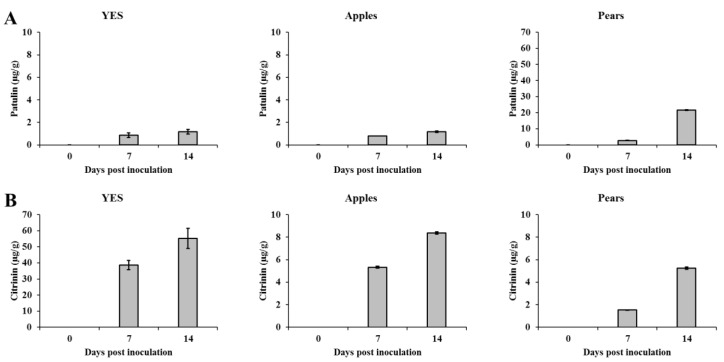
PAT (**A**) and CTN (**B**) production by *P. expansum* Pe-21 strain after 7 and 14 days of growth on YES medium, apples, and pears. Average values of three replicates (±standard error) are reported. Experiments were repeated three times and results of a single representative experiment are shown.

According to the method described above, PAT and CTN were detected and quantified successfully in YES medium and fruits inoculated with *P. expansum*. In YES agar medium, PAT was detected at a concentration of 0.86 µg/g at day 7 postinoculation, which was elevated up to 1.2 µg/g by the end of the experiment (day 14; Figure 1A). In apple fruits colonized by *P. expansum*, PAT was found at the concentrations of 0.8 µg/g and 1.2 µg/g at days 7 and 14 of the experiment, respectively (Figure 1A). Significantly higher levels of mycotoxin were found in pears (up to 22 µg/g; Figure 1A). *P. expansum* can cause significant losses in pear fruits [41]. Spadaro et al. (2008) observed a high incidence of PAT in pear juices suggesting that also pears could be suitable substrates for fungal colonization and PAT contamination [42]. In addition to PAT biosynthesis, our *P. expansum* Pe-21 strain proved to be a good producer of CTN. In YES medium, CTN was produced at levels as high as 55 µg/g (Figure 1B). As has been noted previously, YES agar was shown to be an effective medium for CTN production by *P. expansum* isolates [43]. CTN was detected in apple and pear fruit samples, at levels of 5.3 and 1.5 µg/g by day 7 postinoculation, respectively. As expected, higher CTN levels, ranging from 5.3 to 8.4 µg/g, were found in fruit samples by day 14 of the experiment (Figure 1B).

In conclusion, the rapid and simple HPLC method presented here has been developed and evaluated to determine PAT and CTN in apples and pears. Compared to the majority of studies that described an additional clean-up procedure, in the current work, the developed method was successfully applied to the extractions of mycotoxins from fruit samples and HPLC analysis with no further clean-up and with sufficient sensitivity and selectivity to detect and accurately quantify mycotoxin contents in apples and pears. The main advantage of the current procedure is simultaneous multiple sample handling in a short time period with good recovery rates. The current method also reduced many interfering peaks and enabled us to obtain a clear spectrum with distinct peaks for qualitative and quantitative analysis of PAT and CTN. Thus, the proposed method can be used for routine monitoring of PAT and CTN levels in widely consumed pome fruits and their derived products to keep mycotoxins out of the food supply chain and reduce the hazard to human health.

## 3. Materials and Methods

### 3.1. Fruit and Synthetic Medium Inoculation

The wild-type *Penicillium expansum* Pe-21 strain was grown at 28 °C and maintained on potato dextrose agar (PDA) plates (BD, Franklin Lakes, NJ, USA). Conidia were harvested and adjusted using a hemocytometer to the indicated concentrations. Golden Delicious apples and Spadona pears were obtained from a local supermarket. Fruits were subjected to surface sterilization using 1% sodium hypochlorite solution for 1 min and immediately rinsed in sterile distilled water. A 10 µL conidial suspension containing 10^6^ conidia/mL was injected directly into the sterilized fruits at 2 mm depth. Following inoculation, the fruits were incubated in covered plastic containers at 28 °C for 7 or 14 days. Experiments in a synthetic medium were performed on a solid YES medium (20 g bacto yeast extract, 150 g sucrose, and 15 g bacto agar per liter). Then, 55 mm agar plates containing 10^5^ conidia of each strain were incubated at 28 °C for 7 or 14 days.

### 3.2. Mycotoxin Extraction from Synthetic Medium and Fruits

For fungal cultures grown on YES medium, PAT was extracted from 1 g of the homogenized medium containing fungal mycelium, which was mixed with 2 mL of HPLC grade ethyl acetate (BioLab, Jerusalem, Israel) and placed in an orbital shaker at 150 rpm for 30 min at room temperature. The sample was centrifuged for 10 min at 6000× *g*; the supernatant was transferred to a clean glass tube and was evaporated to dryness under a stream of gaseous nitrogen at 50 °C. The residue was redissolved in 1 mL of the mobile phase (0.02 M ammonium acetate:acetonitrile 9:1 *v*/*v*) and filtered through a 0.22 µM PTFE syringe filter prior to HPLC analysis. CTN was extracted from 1 g of the homogenized YES medium with fungal mycelium using 1 mL of methanol (HPLC grade; BioLab, Jerusalem, Israel). The sample was shaken in an orbital shaker at 150 rpm for 30 min at room temperature and then centrifuged for 10 min at 6000× *g*. The supernatant was filtered through a 0.22 µM PTFE syringe filter and kept at −20 °C prior to HPLC analysis.

For fruit samples, PAT was extracted from 1 g of homogenized apple/pear tissue samples using 2 mL ethyl acetate in a 15 mL glass tube. Each sample was vortexed for at least 5 min; then, 2 mL of 1.5% sodium bicarbonate solution was added, and the sample was vortexed vigorously again for 1 min. Next, 20 µL of acetic acid was added, and after shaking in an orbital shaker at 250 rpm for 5 min, the sample was left to stand for a few minutes at room temperature for phase separation. The upper phase was transferred to a new glass tube and evaporated to dryness under a stream of gaseous nitrogen at 50 °C. The samples were reconstituted in the mobile phase (0.02 M ammonium acetate:acetonitrile 9:1 *v*/*v*) and filtered through a 0.22 µm PTFE syringe filter into a glass injection vial to prepare for the HPLC analysis. CTN was extracted from 1 g of homogenized apple/pear tissue using 2 mL of methanol. Each sample was then vortexed for 5 min and shaken using an orbital shaker at 250 rpm for 30 min before centrifuging at 6000× *g* for 10 min. The aliquots were filtered through a 0.22 µm PTFE membrane syringe filter into a glass injection vial and stored at −20 °C prior to HPLC analysis. 

### 3.3. HPLC Analysis

Both PAT and CTN were quantitatively analyzed by injection of 20 µL filtered samples into a reverse-phase UHPLC system (Waters ACQUITY Arc, FTN-R, Milford, MA, USA) using a Kinetex 3.5 µm XB-C18 (150 × 4.6 mm) column (Phenomenex, Torrance, CA, USA). The column temperature was maintained at 30 °C, and the flow rate was 0.8 and 1 mL/min, respectively. For PAT, the separation was achieved with a mobile phase composed of 0.02 M ammonium acetate:acetonitrile (9:1 *v*/*v*); CTN mobile phase consisted of 1% acetic acid in water and acetonitrile (50:50 *v*/*v*). The PAT peak was detected with a photodiode array (PDA) detector at 276 nm; CTN was detected with a fluorescence detector (331 nm excitation, 500 nm emission). Both mycotoxins were quantified by comparing peak areas with calibration curves of the standard mycotoxins (Fermentek, Jerusalem, Israel).

### 3.4. Validation of Analytical Parameters

Method performance and validation parameters including specificity, linearity (R^2^), the limit of detection (LOD), the limit of quantification (LOQ), and recovery rates were determined according to Commission Regulation (EC) No 401/2006 (European Commission, 2006a) (Table 1 and Table 2). Recovery experiments were performed by spiking blank samples with PAT or CTN at eight different concentrations (Table 1 and Table 2) to ensure the accuracy of the method. The data for recovery were calculated by comparing the absolute responses of PAT/CTN from the spiking samples to the absolute response of the mycotoxin standard solutions. Extraction and analysis were performed as described above. The spiking experiments were performed in triplicate at three different time points.

## Figures and Tables

**Table 1:** Concentration range (µg/g) of calibration curves for analysis of PAT and CTN in YES medium and fruits with corresponding LOD and LOQ (µg/g).

Matrix	PAT (µg/g)			CTN (µg/g)		
	Range	LOD	LOQ	Range	LOD	LOQ
YES medium	0.005–50	0.003	0.009	0.005–50	0.003	0.009
Apple	0.0125–50	0.006	0.018	0.005–50	0.001	0.003
Pear	0.0125–50	0.006	0.018	0.005–50	0.001	0.003

**Table 2:** Mean recoveries and RSD from blank samples spiked with PAT and CTN at different concentrations.

Matrix	PAT		CTN	
	Spiking Levels (µg/g)	Mean Recovery, RSD (%)	Spiking Levels (µg/g)	Mean Recovery, RSD (%)
YES medium	0.005	57.20 (1.73)	0.005	70.99 (0.85)
	0.05	61.86 (3.16)	0.05	83.85 (2.03)
	0.1	67.88 (0.67)	0.1	95.48 (6.95)
	0.5	86.99 (3.95)	0.5	90.99 (3.43)
	1	89.08 (4.70)	1	83.54 (1.33)
	10	83.82 (1.17)	10	88.22 (2.64)
	25	84.64 (1.68)	25	92.93 (4.27)
	50	92.82 (2.97)	50	94.37 (2.28)
Apple	0.0125	55.48 (1.96)	0.005	86.88 (1.60)
	0.05	61.66 (4.35)	0.05	98.91 (7.20)
	0.1	74.79 (6.10)	0.1	101.05 (8.10)
	0.5	84.61 (3.06)	0.5	91.47 (2.83)
	1	78.84 (6.24)	1	89.04 (5.18)
	10	73.63(4.36)	10	89.36 (2.36)
	25	75.07 (9.07)	25	93.82 (1.75)
	50	77.58 (5.66)	50	85.72 (4.74)
Pear	0.0125	57.59 (1.61)	0.005	84.22 (6.33)
	0.05	59.82 (1.07)	0.05	99.68 (5.40)
	0.1	74.55 (2.08)	0.1	99.62 (6.19)
	0.5	83.40 (1.44)	0.5	90.71 (3.21)
	1	97.30 (2.21)	1	87.72 (3.37)
	10	79.87 (4.60)	10	86.92 (3.64)
	25	92.14 (1.94)	25	93.23 (3.77)
	50	94.90 (2.45)	50	93.75 (1.93)

## Data Availability

The data presented in this study are available in this article.

## Supplementary Materials

The following are available online, Figure S1: HPLC chromatograms of PAT and CTN in YES medium, apple and pear fruit samples, Figure S2: Calibration curves for PAT and CTN in YES medium, apple and pear fruit samples.

## Sample Availability

Not available.

## References

[1] Bennett J.W., Klich M. (2003). Mycotoxins. Clin. Microbiol. Rev..

[2] Freire F., da Rocha M. (2016). Impact of mycotoxins on human health. Fungal Metabolites.

[3] Clarke M. (2006). The 1944 patulin trial of the British medical research council. J. R. Soc. Med..

[4] Iha M.H., Sabino M. (2006). Determination of patulin in apple juice by liquid chromatography. J. AOAC Int..

[5] Neri F., Donati I., Veronesi F., Mazzoni D., Mari M. (2010). Evaluation of *Penicillium expansum* isolates for aggressiveness, growth and patulin accumulation in usual and less common fruit hosts. Int. J. Food Microbiol..

[6] Reddy K.R.N., Spadaro D., Lore A., Gullino M.L., Garibaldi A. (2010). Potential of patulin production by *Penicillium expansum* strains on various fruits. Mycotox. Res..

[7] Sadok I., Szmagara A., Staniszewska M.M. (2018). The validated and sensitive HPLC-DAD method for determination of patulin in strawberries. Food Chem..

[8] Heussner A.H., Dietrich D.R., O’Brien E. (2006). In vitro investigation of individual and combined cytotoxic effects of ochratoxin A and other selected mycotoxins on renal cells. Toxicol. Vitr..

[9] Schumacher D.M., Metzler M., Lehmann L. (2005). Mutagenicity of the mycotoxin patulin in cultured Chinese hamster V79 cells, and its modulation by intracellular glutathione. Arch. Toxicol..

[10] Boussabbeh M., Salem I.B., Prola A., Guilbert A., Bacha H., Abid-Essefi S., Lemaire C. (2015). Patulin induces apoptosis through ROS-mediated endoplasmic reticulum stress pathway. Toxicol. Sci..

[11] Moake M.M., Padilla-Zakour O.I., Worobo R.W. (2005). Comprehensive review of patulin control methods in foods. Compr. Rev. Food Sci. Food Saf..

[12] Zouaoui N., Mallebrera B., Berrada H., Abid-Essefi S., Bacha H., Ruiz M.J. (2016). Cytotoxic effects induced by patulin, sterigmatocystin and beauvericin on CHO-K1 cells. Food Chem. Toxicol..

[13] World Health Organization (WHO) (1995). 44th Report of the Joint FAO/WHO Expert Committee on Food Additives.

[14] EU (2006). Commission Regulation (EC) No (1881/2006) of 19 December 2006 Setting Maximum Levels for Certain Contaminants in Foodstuffs.

[15] Hetherington A.C., Raistrick H. (1931). Studies in the biochemistry of micro-organisms. On the Production and Chemical Constitution of a New Yellow Colouring Matter, Citrinin, Produced from Glucose by Penicillium citrinum.

[16] Xu B.J., Jia X.Q., Gu L.J., Sung C.K. (2006). Review on the qualitative and quantitative analysis of the mycotoxin citrinin. Food Control..

[17] (2012). Scientific opinion on the risks for public and animal health related to the presence of citrinin in food and feed. EFSA J..

[18] Ostry V., Malir F., Ruprich J. (2013). Producers and important dietary sources of ochratoxin A and citrinin. Toxins.

[19] López Sáncheza P., de Nijsa M., Spanjerb M., Pietric A., Bertuzzic T., Starski A., Postupolski J., Castellari M., Hortós M. (2017). Generation of occurrence data on citrinin in food. EFSA Support. Publ..

[20] Hanika C., Carlton W.W., Tuite J. (1983). Citrinin mycotoxicosis in the rabbit. Food Chem. Toxicol..

[21] Bilgrami K.S., Sinha S.P., Jeswal P. (1988). Nephrotoxic and hepatoxic effects of citrinin in mice (*Mus musculus*). Proc. Indian Natl. Sci. Acad..

[22] Speijers G.J.A., Speijers M.H.M. (2004). Combined toxic effects of mycotoxins. Toxicol. Lett..

[23] Jeswal P. (1996). Citrinin-induced chromosomal abnormalities in the bone marrow cells of *Mus musculus*. Cytobios.

[24] Pfohl-Leszkowicz A., Petkova-Bocharova T., Chernozemsky I.N., Castegnaro M. (2002). Balkan endemic nephropathy and associated urinary tract tumours: A review on aetiological causes and the potential role of mycotoxins. Food Addit. Contam..

[25] International Agency for Research on Cancer (IARC) (1993). Monographs on the Evaluation of Carcinogenic Risks to Humans; Some Naturally Occurring Substances: Food Items and Constituents, Heterocyclic Aromatic Amines and Mycotoxins.

[26] Vidal A., Ouhibi S., Ghali R., Hedhili A., De Saeger S., De Boevre M. (2019). The mycotoxin patulin: An updated short review on occurrence, toxicity and analytical challenges. Food Chem. Toxicol..

[27] Atapattu S.N., Poole C.F. (2020). Recent advances in analytical methods for the determination of citrinin in food matrices. J. Chromatogr. A.

[28] Guo B.Y., Wang S., Ren B., Li X., Qin F., Li J. (2010). Citrinin selective molecularly imprinted polymers for SPE. J. Sep. Sci..

[29] Meister U. (2004). New method of citrinin determination by HPLC after polyamide column clean-up. Eur. Food Res. Technol..

[30] Wang M., Jiang N., Xian H., Wei D.Z., Shi L., Feng X.Y. (2016). A single-step solid phase extraction for the simultaneous determination of 8 mycotoxins in fruits by ultra-high performance liquid chromatography tandem mass spectrometry. J. Chromatogr. A.

[31] Li Y., Zhou Y.C., Yang M.H., Ou-Yang Z. (2012). Natural occurrence of citrinin in widely consumed traditional Chinese food red yeast rice, medicinal plants and their related products. Food Chem..

[32] Desmarchelier A., Mujahid C., Racault L., Perring L., Lancova K. (2011). Analysis of patulin in pear- and apple-based foodstuffs by liquid chromatography electrospray ionization tandem mass spectrometry. J. Agric. Food Chem..

[33] Marsol-Vall A., Delpino-Rius A., Eras J., Balcells M., Canela-Garayoa R. (2014). A Fast and reliable UHPLC-PDA method for determination of patulin in apple food products using QuEChERS extraction. Food Anal. Methods.

[34] Sadok I., Stachniuk A., Staniszewska M. (2019). Developments in the monitoring of patulin in fruits using liquid chromatography: An overview. Food Anal. Methods.

[35] EU (2006). Commission Regulation (EC) No 401/2006 of 23 February 2006 laying down the methods of sampling and analysis for the official control of the levels of mycotoxins in foodstuffs. Off. J. Eur. Union.

[36] Valle-Algarra F.M., Mateo E.M., Gimeno-Adelantado J.V., Mateo-Castro R., Jiménez M. (2009). Optimization of clean-up procedure for patulin determination in apple juice and apple purees by liquid chromatography. Talanta.

[37] De Clercq N., Van Pamel E., Van Coillie E., Vlaemynck G., Devlieghere F., De Meulenaer B., Daeseleire E. (2016). Optimization and validation of a method without alkaline clean-up for patulin analysis on apple puree agar medium (APAM) and apple products. Food Anal. Methods.

[38] Meerpoel C., Vidal A., di Mavungu J.D., Huybrechts B., Tangni E.K., Devreese M., Croubels S., De Saeger S. (2018). Development and validation of an LC–MS/MS method for the simultaneous determination of citrinin and ochratoxin a in a variety of feed and foodstuffs. J. Chromatogr. A.

[39] Martins M.L., Gimeno A., Martins H.M., Bernardo F. (2002). Co-occurrence of patulin and citrinin in Portuguese apples with rotten spots. Food Addit. Contam..

[40] Tolgyesi A., Stroka J., Tamosiunas V., Zwickel T. (2015). Simultaneous analysis of alternaria toxins and citrinin in tomato: An optimised method using liquid chromatography-tandem mass spectrometry. Food Addit. Contam.Part. A.

[41] Zhang H., Wang L., Dong Y., Jiang S., Zhang H., Zheng X. (2008). Control of postharvest pear diseases using *Rhodotorula glutinis* and its effects on postharvest quality parameters. Int. J. Food Microbiol..

[42] Spadaro D., Garibaldi A., Gullino M.L. (2008). Occurrence of patulin and its dietary intake through pear, peach, and apricot juices in Italy. Food Addit. Contam. Part B.

[43] Santos I.M., Abrunhosa L., Venâncio A., Lima N. (2002). The effect of culture preservation techniques on patulin and citrinin production by *Penicillium expansum* link. Lett. Appl. Microbiol..

