# Supplementary material for: Rapid Detection and Quantification of Patulin and Citrinin Contamination in Fruits
Source: Molecules. 2021 Jul 27;26(15):4545. doi: 10.3390/molecules26154545 (PMC8348754; doi:10.3390/molecules26154545)
Supplement: Supplementary file 1 [file molecules-26-04545-s001.zip › molecules-1285486-Supplementary-final.pdf]

Supplementary material

HPLC chromatogram of PAT in YES medium

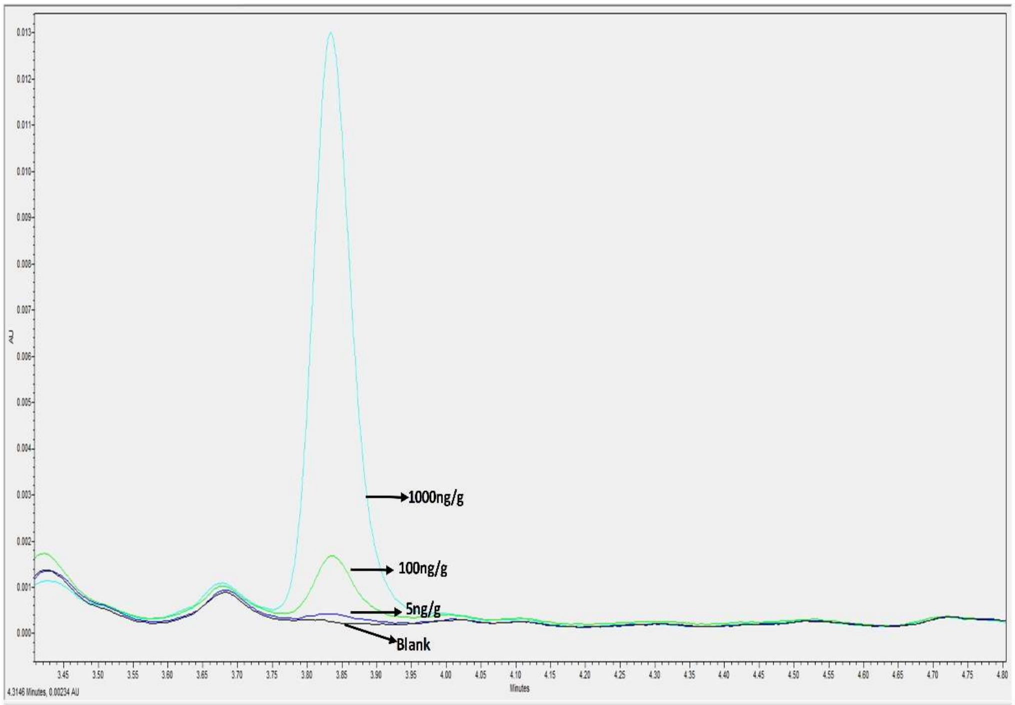

HPLC chromatogram of PAT in apple

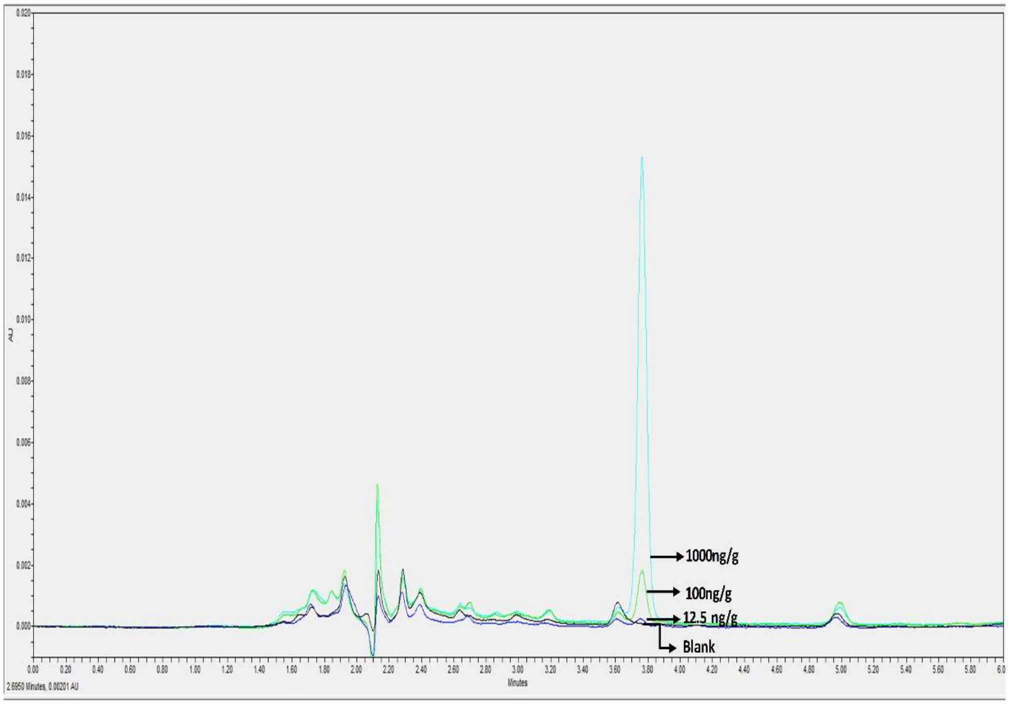

HPLC chromatogram of PAT in pear

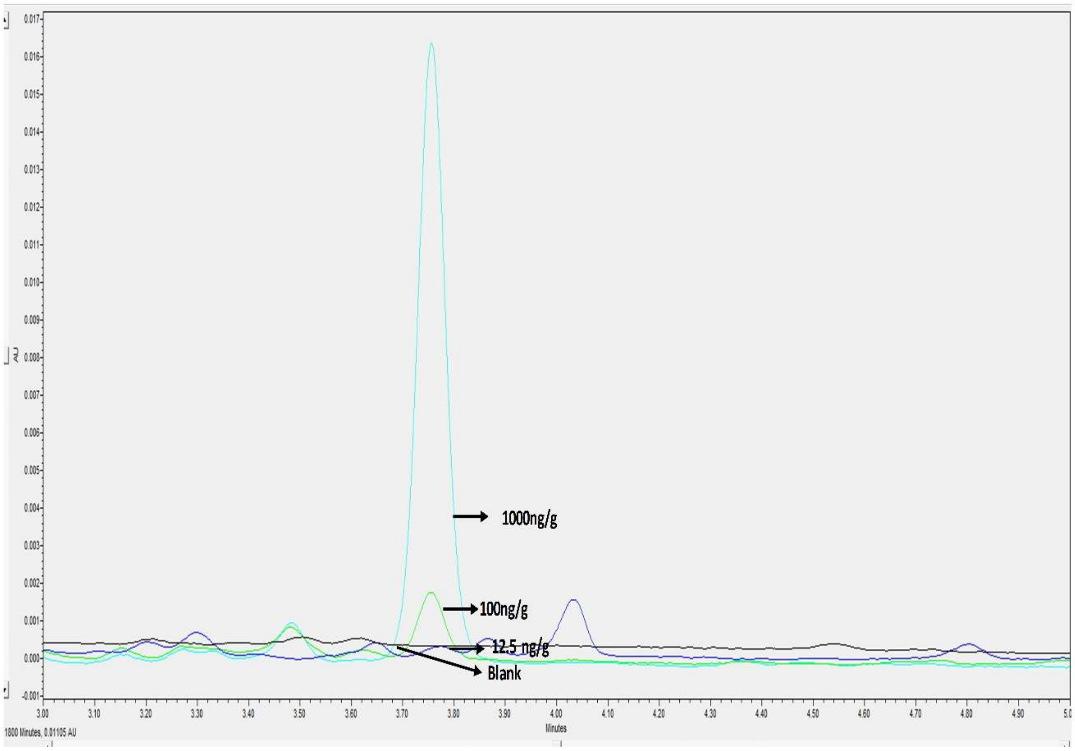

HPLC chromatogram of CTN in YES medium

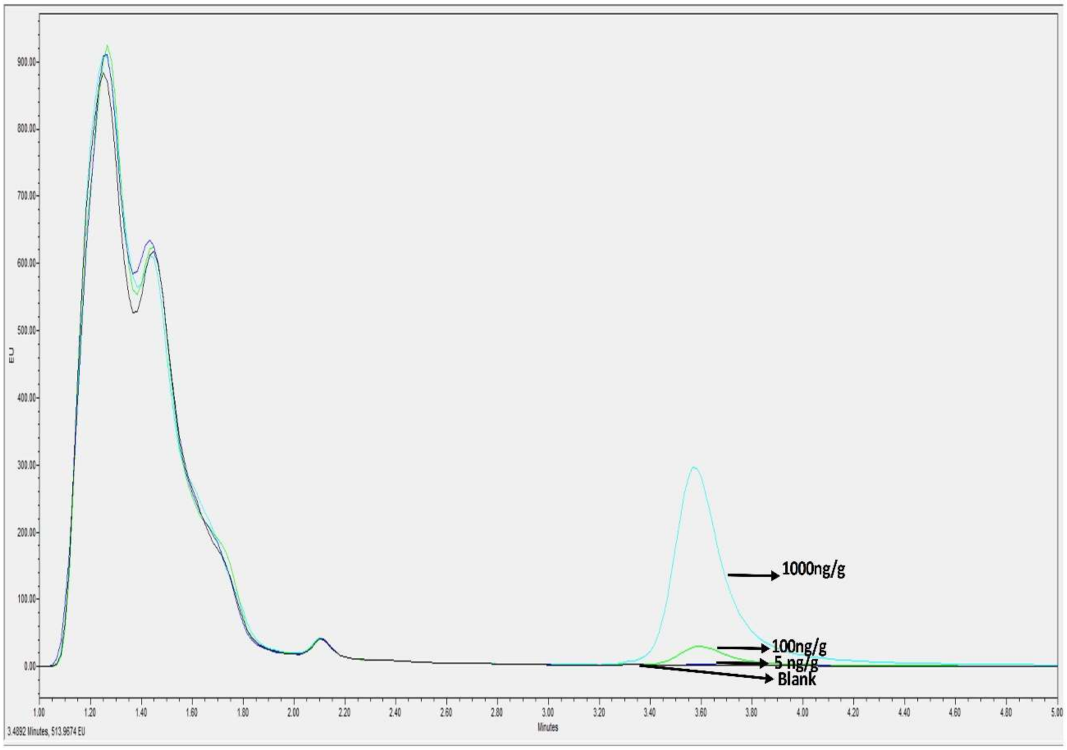

HPLC chromatogram of CTN in apple

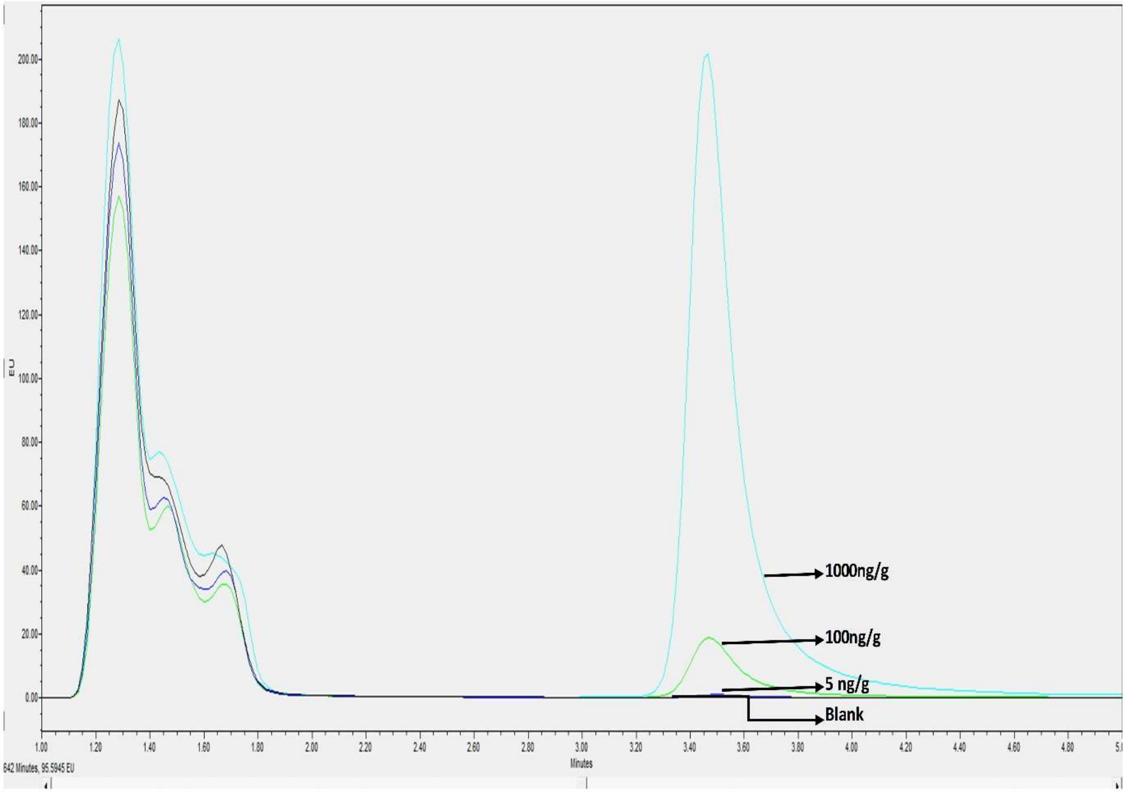

HPLC chromatogram of CTN in pear

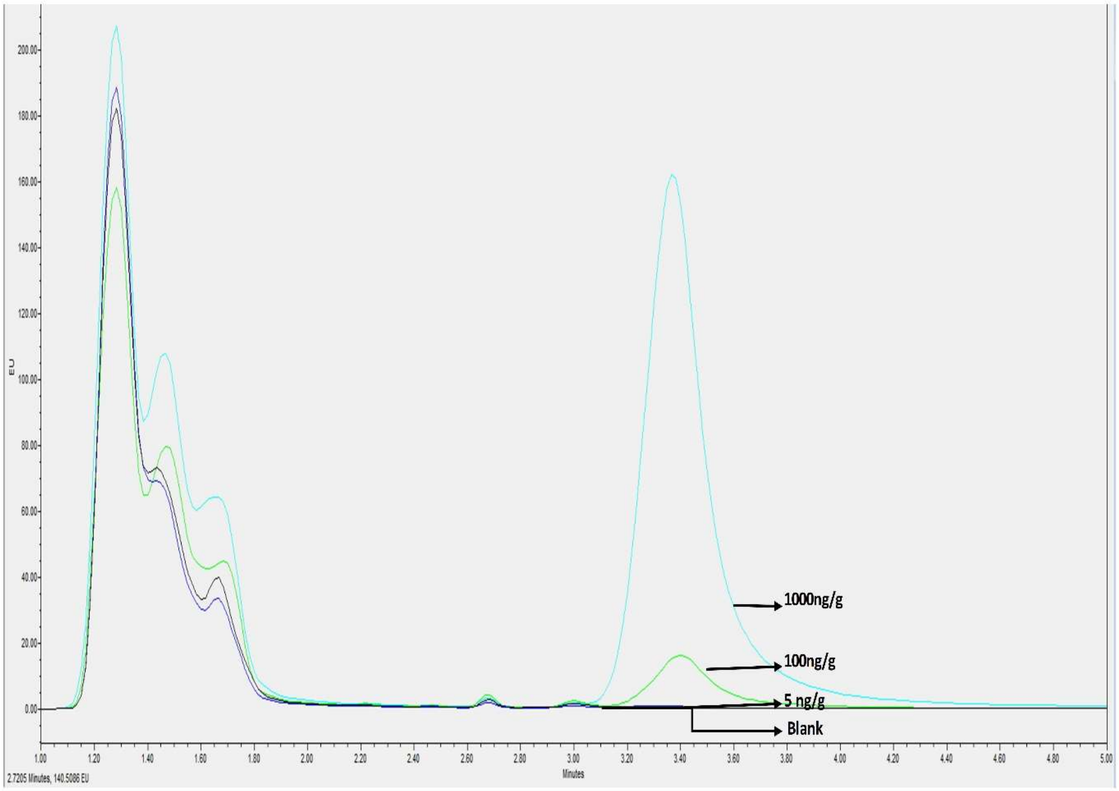

Figure S1. HPLC chromatograms of PAT and CTN in YES medium, apple and pear fruit samples

YES medium spiked with PAT

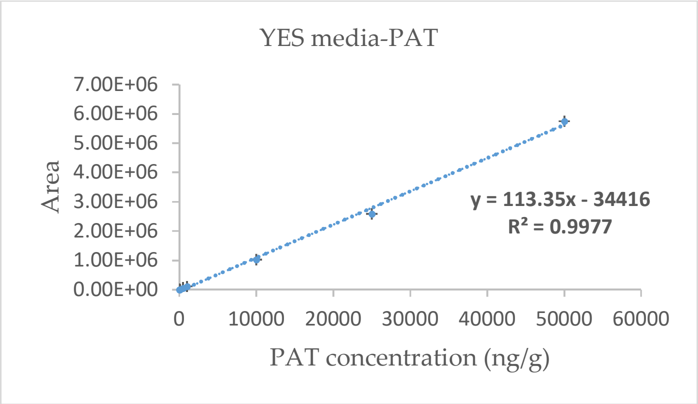

Apple spiked with PAT

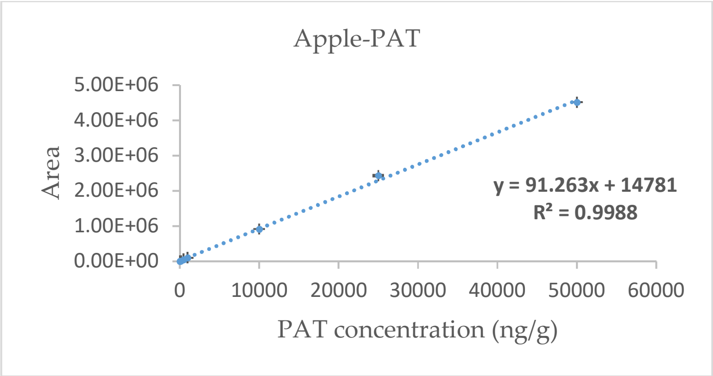

Pear spiked with PAT

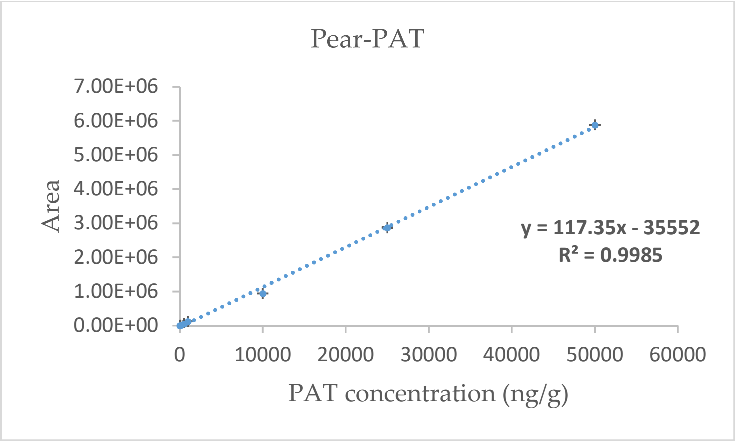

YES medium spiked with CTN

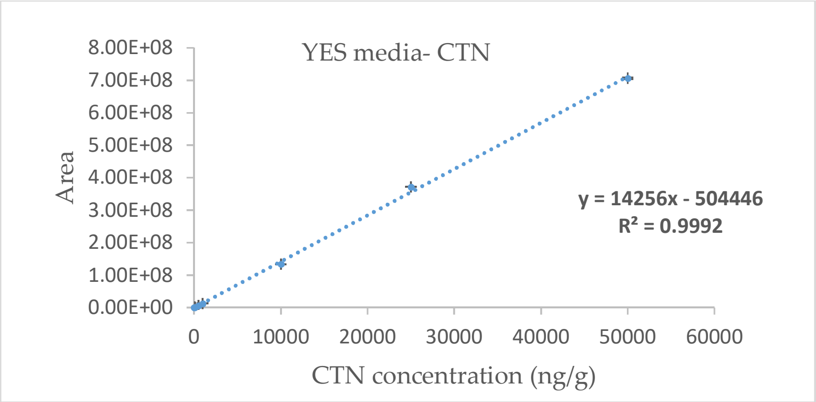

Apple spiked with CTN

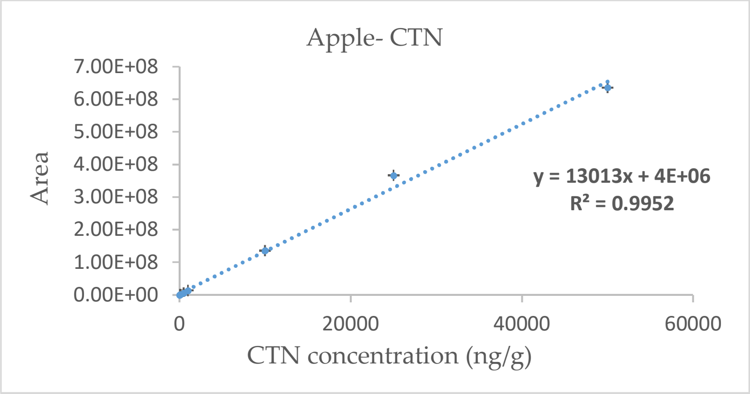

Pear spiked with CTN

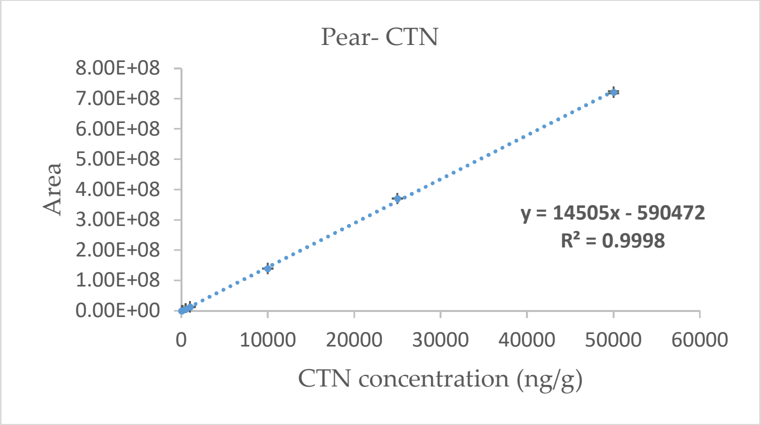

Figure S2. Calibration curves for PAT and CTN in YES medium, apple and pear fruit samples
